# Trusted but isolated: how perceived trust from leaders leads to workplace exclusion through being the target of envy

**DOI:** 10.3389/fpsyg.2025.1680581

**Published:** 2025-09-25

**Authors:** Linjia Song, Kai Yao, Lingyu Li

**Affiliations:** ^1^Faculty of Humanities and Tourism, Yibin Vocational and Technical College, Yibin, China; ^2^Faculty of Business and Finance Management, Rattana Bundit University, Bangkok, Thailand; ^3^Jinhua University of Vocational Technology, Jinhua, China

**Keywords:** inclusive leadership, perceived trust from leaders, workplace exclusion, being the target of envy, social comparison theory, perceived competitive climate, multicultural teams

## Abstract

This study explores the unintended interpersonal consequences of perceived trust from leaders—a core element of inclusive leadership—within multicultural team settings. While leader trust is typically associated with positive outcomes, our research reveals its paradoxical role in fostering workplace exclusion. Drawing on social comparison theory and using a two-study design (a contextual experiment and a multi-source field survey), we examine how perceived trust from leaders can lead to being the target of envy among coworkers and trigger workplace exclusion behaviors. Furthermore, the perceived competitive climate amplifies these effects, highlighting the boundary conditions under which inclusive leadership practices may unintentionally harm team dynamics. The findings provide novel insights into the “dark side” of inclusive leadership, offering practical guidance for managing trust and team competitiveness in culturally diverse organizations. Robustness checks addressing stable individual differences yielded the same pattern of results.

## 1 Introduction

With the rapid development of intelligent technology, simple and repetitive manual labor has gradually been replaced by complex and flexible mental labor. Accordingly, the management style that emphasizes supervision and control has also begun to change to a management style based on autonomy and trust. In this context, making employees feel the trust of their leaders is considered an effective management strategy for leaders (Wang and Zhang, 2016). Perceived trust from leaders refers to the degree to which employees perceive that their leaders are willing to take risks for their actions (Baer et al., 2015). At present, the research on the outcome variables of Perceived trust from leaders is mainly divided into two parts: one part explores the impact of Perceived trust from leaders on employees from the perspective of employees themselves, including the impact on employees’ self-concept, work attitude, work behavior, work performance, etc. For example, Lau et al. (2014) found that Perceived trust from leaders can indirectly promote employees’ organizational citizenship behavior and improve work performance by enhancing employees’ organizational self-esteem; Wang and Zhang (2016) and Chen et al. (2020) found that Perceived trust from leaders can also lead to negative consequences such as emotional exhaustion and counterproductive behavior. Another part of the research explores the impact of perceived trust from leaders on the relationship between employees and leaders from the perspective of the relationship between employees and leaders. For example, Skiba and Wildman (2019) found that perceived trust from leaders would enhance the exchange relationship between employees and leaders and increase employees’ sense of obligation to their leaders; Deng and Wang (2009) showed that perceived trust from leaders positively affects employees’ satisfaction with their leaders and makes them more loyal to their leaders. Although the above studies provide a certain theoretical and empirical basis for understanding the role of perceived trust from leaders in organizations, these studies have neglected to explore the influence mechanism of trust from leaders from the perspective of inter-employee relationships. This limits researchers’ ability to comprehensively and balancedly assess the impact of perceived trust from leaders in organizations, nor is it conducive to managers effectively dealing with issues related to trust between superiors and subordinates and inter-employee relationships.

According to previous research, studying the issue of Perceived trust from leaders from the perspective of inter-employee relationships is not without basis. Some studies have shown that Perceived trust from leaders may affect the interaction between employees and their colleagues. For example, since leader trust is scarce in organizations (Chen et al., 2020; Graen and Uhl-Bien, 1995), and leader trust in employees also means that leaders rely more on and allocate more resources to these employees (Mayer et al., 1995), leader trust is not only related to the leader and the trusted employee, but also closely related to the interests of other colleagues in the organization. Employees with stronger leader relationships are more likely to be targets of upward social comparison and envy from colleagues (Sun et al., 2020), which is not conducive to interaction with colleagues (Koopman et al., 2020). Prior research shows that trust in leaders is a pivotal mechanism shaping employee attitudes and behaviors, predicting commitment, collaboration, productivity, and well-being at work. Recent syntheses also indicate robust links from leadership trust to performance-relevant outcomes across leadership styles. Building on this stream, we argue that high trust in a focal employee can act as a visible status cue that triggers social comparison processes among coworkers. These preliminary studies have laid an important foundation for this paper to further explore how leader trust affects inter-colleague relationships.

Workplace envy research distinguishes between reactions of the envious and reactions of the envied. Coworkers who envy a leader-trusted employee may respond with withdrawal or ostracizing the target, whereas the envied target may cope by distancing from peers. Our focus is on the latter—how feeling envied shapes the target employee’s exclusionary responses—while acknowledging that coworkers’ ostracism toward the trusted target is a conceptually distinct phenomenon.

**Figure 1:**
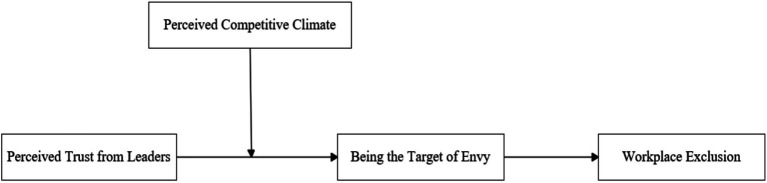
Theoretical model.

Based on this, this study will explore why, how, and when perceived trust from leaders affects the interaction between employees and colleagues from the perspective of inter-employee relationships, based on social comparison theory and related literature on envy (as shown in Figure 1). According to social comparison theory (Festinger, 1954), when an individual possesses resources that others desire but lack, the individual will become the object of upward social comparison by others, and upward social comparison between others and oneself will make the individual feel envied (Liu et al., 2018). Because Perceived trust from leaders is scarce (Chen et al., 2020; Graen and Uhl-Bien, 1995), employees who perceive trust from leaders may feel that they have become the object of upward social comparison by their colleagues, possessing things that their colleagues desire but lack, thus feeling envied (Liu et al., 2018). At the same time, in order to avoid being hurt by the envious, the envied employee may adopt a subtle, passive avoidance interpersonal coping style (Liu et al., 2018), ignoring and alienating the surrounding colleagues, making the colleagues feel excluded. Based on previous research, since an individual’s social comparison process and its impact are influenced by the team environment they are in Liu et al. (2017), we propose that the relationship between trust from leaders, employee envy, and workplace exclusion is also influenced by the team environment. Considering that Perceived trust from leaders is an important resource with scarcity, employees may engage in social comparison and competition for it. Therefore, this study incorporates the perception of team competition climate as an important contextual factor into the model. When employees perceive a strong Perceived competitive climate within their team, they are more likely to believe that they will become the target of upward social comparison by their colleagues, feel envied, and therefore increase their workplace exclusionary behaviors toward colleagues (e.g., ignoring and alienating colleagues).

This study contributes to the literature on perceived trust, envy, and workplace exclusion in four key areas: First, this study explores the impact of perceived trust from a supervisor from the perspective of employee-colleague relationships, expanding the research perspective on the outcome variable of perceived trust from a supervisor; Second, by examining the mechanism by which perceived trust from a supervisor influences employee workplace exclusionary behavior, this study reveals the “black box” of how perceived trust from a supervisor influences employee-colleague relationships, further enriching research on the “dark side” of perceived trust from a supervisor; Third, this study examines envy as a mediating variable, expanding research on the antecedents and consequences of envy; Fourth, we investigate “why individuals exclude others” from the perspective of the excluder, enriching research on the antecedents of workplace exclusion from this perspective.

The study conceptually differentiate workplace ostracism—being ignored or excluded by others—from the target’s exclusionary behavior, namely the target’s deliberate withdrawal or avoidance toward coworkers as a coping response to feeling envied. The former is other-directed mistreatment experienced by the target; the latter is target-initiated defensive distancing.

## 2 Theoretical basis and research hypotheses

### 2.1 Social comparison theory and perceived trust from leaders

Social comparison theory holds that individuals tend to accurately evaluate themselves, and these evaluations rely on social comparisons with others (Festinger, 1954). By comparing themselves with others in various aspects (including abilities, opinions, and authority), individuals can discover their true selves and evaluate their current environment based on the relevant information from social comparisons (Tao and Hu, 2015). Who will individuals choose as reference objects for social comparison? Social comparison theory holds that individuals choose reference objects for social comparison based on the availability of information in the environment and the similarity of the reference objects. When individuals have easier access to relevant information about certain objects or are more similar to certain objects, they are more likely to choose them as reference objects for social comparison. Since colleagues in the same team are often similar and it is easier for them to obtain information about each other, colleagues in the same team are often important reference objects for social comparison (Oldham et al., 1986).

Perceived trust from leaders reflects the extent to which employees believe their supervisors trust them, signaling the quality of leader–subordinate relationships. Within teams, leaders tend to develop differentiated trust relationships with individual subordinates (Erdogan and Bauer, 2010). Employees who are trusted from leaders are often regarded as “insiders” within the leader–member exchange framework. Compared to “outsiders,” leaders are more emotionally connected to insiders, frequently assigning them critical tasks and providing greater support (Zheng, 1999). Such preferential treatment offers insiders a competitive edge in securing organizational resources and career development opportunities. Consequently, employees place high value on leader trust and often regard it as a key criterion in social comparisons with their colleagues (Tao and Hu, 2015). In this study, we distinguish between *envy* (an emotion felt by individuals toward others) and being the target of envy (an individual’s being the target of envy by others). The current research focuses on the latter.

### 2.2 The interactive effect of perceived trust from leaders and perceived competitive climate on being the target of envy

Being the target of envy refers to an individual’s perception that others envy them—essentially a cognitive appraisal of others’ envy from the perspective of the envied person (Liu et al., 2018). When individuals engage in social comparison and perceive that they possess valued attributes or resources that others lack—such as trust from leadership—they are more likely to feel envied. This experience is shaped by both leadership behaviors and broader organizational factors (Liu et al., 2018).

Perceived trust from leaders not only signifies a strong leader–employee relationship but also often translates into tangible benefits, such as favorable task assignments, increased promotion opportunities, and higher performance evaluations (Mayer et al., 1995). As a result, leader trust becomes a desirable and scarce resource that employees compete for, providing fertile ground for social comparison. When an employee is perceived to be trusted by leadership, the ensuing comparison with colleagues may lead them to believe that they are receiving more organizational resources than others. Given the limited nature of leader trust (Chen et al., 2020; Graen and Uhl-Bien, 1995), this perceived advantage implies that other team members are receiving less. Consequently, the trusted employee may come to believe that they are the target of envy—envied for possessing what others aspire to but do not have (Liu et al., 2018).

At the same time, the feeling of being the target of envy by Perceived trust from leaders is stronger in a competitive team climate. Perceived competitive climate is an employee’s individual perception of the competition among colleagues for compensation, recognition, and status (Brown et al., 1998), which reflects the employee’s perception of whether the team climate encourages colleagues to compete for resources (Fletcher et al., 2008). Research shows that a strong Perceived competitive climate will strengthen the individual’s social comparison process and have a significant impact on individual attitudes (Fletcher et al., 2008). In a highly competitive team climate, employees perceive greater comparison and competition among colleagues (Brown et al., 1998). In a Perceived competitive climate, employees who feel trusted by their leaders will compare more with their colleagues around them, and thus more strongly believe that they have the “envy object” that their colleagues want but lack - Perceived trust from leaders, and thus have a stronger feeling of being the target of envy by their colleagues. Conversely, when employees perceive a weak Perceived competitive climate within the team, they will feel that the degree of comparison and competition among colleagues is weaker. At this time, even if employees feel trusted by their leaders, they will rarely make social comparisons with their colleagues. Recent reviews and meta-analytic evidence consolidate that workplace envy is rooted in social comparison and yields distinct outcomes for the envious versus the envied actor, with leadership signals often amplifying comparison salience (Duffy et al., 2021; Li et al., 2023). New studies further map antecedents and boundary conditions in leadership contexts, showing how leader trust and recognition function as visible status cues that shape peer dynamics and downstream behaviors (Legood et al., 2021; Dirks and Ferrin, 2002). Building on these insights, we position leader trust as a comparison cue and focus on the envied target’s behavioral responses. Therefore, it is difficult for the perception of being trusted by their leaders to be transformed into a feeling of being the target of envy by their colleagues. Based on the above analysis, this study proposes the following hypotheses:

*Hypothesis 1*: Perceived trust from leaders and Perceived competitive climate interactively influence being the target of envy. Specifically, the stronger the Perceived competitive climate, the stronger the positive impact of perceived trust from leaders on Being the target of envy.

### 2.3 Being the target of envy and workplace exclusionary behavior toward colleagues

Being the target of envy by colleagues can bring employees many negative experiences such as unhappiness, anxiety, worry, and stress, because being the target of envy by colleagues means that employees may face rejection, hostility and social destruction from colleagues (Liu et al., 2018; Liu et al., 2019). Therefore, the motivation to reduce or avoid the negative impact of other colleagues’ envy provides the driving force for individuals to adopt strategies to deal with others’ envy (Liu et al., 2018). Since among the many coping strategies available, “the object of envy” such as Perceived trust from leaders is difficult to share with other colleagues, avoiding contact and escaping may be a more effective strategy (Liu et al., 2018). It is worth noting that the behavior of employees who are envied to avoid contact and escape other colleagues (envious people) is likely to be understood by other colleagues as a form of exclusion. According to Anderson (2009), exclusion is a conscious and active avoidance behavior, the purpose of which is to isolate or exclude a person or organization (Ferris et al., 2008). Unlike other negative interpersonal interactions, such as bullying, verbal abuse, and physical conflict, exclusion is usually manifested as silent behaviors such as ignoring, disregarding, and avoiding eye contact and verbal communication (Robinson et al., 2013). Although avoidance and evasion are merely a way for the envied employee to avoid threats and harm, they encompass the content of workplace exclusion to a certain extent. Therefore, in the eyes of other colleagues, the envied employee’s avoidance and evasion are likely to be excluding themselves. Based on the above analysis, this study proposes the following hypotheses:

*Hypothesis 2*: Being the target of envy positively affects employees' workplace exclusion of colleagues.

Building upon H1 and H2, we propose a moderated mediation model wherein perceived competitive climate amplifies the indirect effect of perceived trust from leaders on workplace exclusion through being the target of envy. Workplace ostracism refers to being ignored or excluded by others—an other-perpetrated mistreatment experienced by the focal employee (Ferris et al., 2008; Williams, 2007; Williams and Nida, 2022). In contrast, our interest lies in the envied target’s exclusionary behavior—target-initiated defensive withdrawal or avoidance toward coworkers as a coping response to feeling envied. Keeping these constructs separate avoids contamination between being excluded by others and choosing to distance oneself and aligns the theorized mechanism with actor-initiated behavior.

Although our theorizing centers on the envied target’s exclusionary coping, we operationalize a closely related downstream outcome—workplace ostracism experienced by the target—to capture relationship deterioration around the envied employee. Ostracism is other-perpetrated, whereas the target’s exclusionary behavior is actor-initiated; we treat ostracism as a conservative indicator of relational distancing that may co-occur with or follow the target’s defensive withdrawal (Ferris et al., 2008; Williams, 2007; Williams and Nida, 2022). We therefore test:

*Hypothesis 3*: Feeling envied will be positively associated with workplace ostracism experienced by the target.

### 2.4 Research design

This paper tests the theoretical model using a multi-study design and multi-sample approach, encompassing an experimental study (Study 1) and a questionnaire survey (Study 2). Study 1 will test Hypothesis 1 through a contextual experiment, establishing a causal relationship between Perceived trust from leaders and perceived competitive climate and Being the target of envy. Study 2 will further test the overall research model using a multi-source questionnaire survey, thereby expanding the external validity of this study.

## 3 Study 1

### 3.1 Research methods

#### 3.1.1 Research sample

Study 1 was a situational experiment. A total of 235 full-time employees of companies were recruited through the alumni network to participate in the experiment. Each participant received a reward of 20 baht after completing the experiment. During the experiment, participants were asked to describe the main content of the experimental situation. After deleting samples whose descriptions did not match the experimental materials, this study ultimately obtained 214 valid samples (effective recovery rate of 91.1%). Among them, 73 were male (34.1%) and 141 were female (65.9%). The average age was 26.51 years (SD = 5.18) and the average length of service was 2.89 years (SD = 4.98). Fourteen participants (6.6%) had a college degree or below, 130 (60.7%) had a bachelor’s degree, 65 (30.4%) had a master’s degree, and 5 (2.3%) had a doctorate. Forty-one participants (19.2%) worked in the information technology industry, 28 (13.1%) worked in the education and training industry, and 15 (15%) worked in the manufacturing industry (7.0%), and 130 people from other industries (accounting for 60. 7%).

#### 3.1.2 Experimental design and procedures

The experiment employed a 2 × 2 between-subjects factorial design, manipulating two independent variables: perceived trust from leaders (high vs. low) and Perceived competitive climate (high vs. low), resulting in four distinct experimental conditions. To ensure random allocation of participants into these four groups, we utilized the *Randomizer* function in Typeform, an online survey platform commonly adopted in academic settings in Thailand for its flexibility and logic control features. This function allows participants to be randomly and automatically assigned to one of the four experimental scenarios, thereby eliminating selection bias and enhancing internal validity. Specifically, 50 participants were assigned to the “high perceived trust × high Perceived competitive climate” condition, 53 to the “high perceived trust × low Perceived competitive climate” condition, 55 to the “low perceived trust × high Perceived competitive climate” condition, and 56 to the “low perceived trust × low Perceived competitive climate” condition. The manipulation materials for perceived trust were adapted from the Perceived trust from leaders scenarios developed by Baer et al. (2021), while the materials for Perceived competitive climate were constructed based on the definition and measurement criteria proposed by Brown et al. (1998). At the beginning of the experiment, participants read a scenario describing a fictional employee named A and were instructed to imagine themselves as the protagonist. To ensure comprehension, they were asked to briefly recall the content. They then completed a questionnaire measuring envy, manipulation checks, and demographic variables.

Manipulation of perceived trust from leaders. In the experimental group (high Perceived trust from leaders), participants read the following text: “You feel that boss has a high level of trust in you. He often relies on your judgment on work-related issues, allowing you to play a role in tasks that are important to him, and he does not believe it’s necessary to supervise your work. Boss also shares his opinions on sensitive issues with you, even when they are unpopular. Furthermore, when you ask him why your work went wrong, he’s honest with you, even if he bears some responsibility.” In the control group (low Perceived trust from leaders), participants read the following text: “You feel that boss has a low level of trust in you. The lead relies solely on his own judgment on work-related issues, requires you to follow his guidance to solve problems that are important to him, and he believes it’s necessary to supervise your work. Furthermore, Boss rarely shares his opinions on sensitive issues with you. Furthermore, when you ask him why your work went wrong, he rarely tells you candidly.”

Manipulation of the perceived competitive climate. In the experimental group (high Perceived competitive climate), participants read the following text: “You find the Perceived competitive climate within your department to be intense. Not only does your boss frequently compare your work results with those of other colleagues, but your colleagues also frequently compare their performance with yours. Everyone is obsessed with achieving top performance. Furthermore, the degree of recognition you receive within the department is determined by your performance ranking.” In the control group (low Perceived competitive climate), participants read the following text: “You find the Perceived competitive climate within your department to be less intense. Your boss rarely compares your work results with those of other colleagues, and your colleagues rarely compare their performance with yours. Few people are obsessed with achieving top performance. Furthermore, the degree of recognition you receive within the department is not determined by your performance ranking.”

Participants were randomly assigned to conditions, which balances stable dispositions such as narcissism or neuroticism across groups. Balance checks showed no between-condition differences on baseline demographics and affectivity proxies (all *p*s > 0.10). We therefore interpret treatment effects as independent of stable personality differences.

#### 3.1.3 Measurement tools

The English scales used in this study were established in previous research, and their reliability and validity have been repeatedly verified in Thai contexts. The scales were translated into Thai using a standard translation-back translation procedure. All variables were statistically analyzed using a 5-point Likert scale, ranging from “1 = strongly disagree” to “5 = strongly agree.”

##### 3.1.3.1 Being the target of envy

In this study, we distinguish between envy and being the target of envy. While envy reflects the emotion one feels toward others, being the target of envy refers to one’s being the target of envy by others. This study focuses on the latter. The scale developed by Lange et al. (2020) was used, which consists of three items and was adjusted according to the experimental context. For example, “I am A, and I feel that my colleagues envy me.” Participants answered the questions based on their current feelings. The Cronbach’s α coefficient of this scale in this study was 0.92.

##### 3.1.3.2 Manipulation test

Perceived trust from leaders scale developed by Baer et al. (2015) was used to conduct a manipulation test of Perceived trust from leaders. The scale consists of 10 items, which were adjusted according to the experimental context. For example, “My boss will let me play a role in work that is important to him.” Participants answered the questions based on their current feelings. The Cronbach’s α coefficient of this scale in this study was 0.94. For the manipulation test of Perceived competitive climate, the Perceived competitive climate scale developed by Brown et al. (1998) was used. It consists of 4 items. For example, “My colleagues often compare their work performance with mine.” Participants answered the questions based on their current feelings. The Cronbach’s α coefficient of this scale in this study was 0.95.

### 3.2 Research results

#### 3.2.1 Manipulation test

The results of the manipulation test showed that the experimental group’s Perceived trust from leaders scores (*M* = 3.40, SD = 0.59) were significantly higher than those of the control group (*M* = 1.91, SD = 0.64), *t* (212) = 17.72, *p* < 0.001, Cohen’s d = 2.42. Furthermore, the high-Perceived competitive climate group’s scores (*M* = 4.10, SD = 0.74) were significantly higher than those of the low-Perceived competitive climate group (*M* = 2.19, SD = 1.02), *t* (212) = 15.60, *p* < 0.001, Cohen’s d = 2.14. Therefore, the manipulation of both variables in this experimental study was successful.

#### 3.2.2 Hypothesis testing

The descriptive statistics and correlation analysis results are shown in Table 1. Hypothesis 1 was tested using linear regression analysis, and the results are shown in Table 2.

**Table 1:** Means, standard deviations, and correlation coefficients of variables in Study 1.

Variable	*M*	*SD*	1	2	3
1. Manipulation of perceived trust from leaders	0.48	0.50	–		
2. Manipulation of perceived competitive climate	0.49	0.50	−0.01	–	
3. Being the target of envy	2.61	0.98	0.15*	0.26***	–

*n* = 214; Manipulation of Perceived Trust from Leaders: 0 = control group, 1 = experimental group; Manipulation of Perceived competitive climate: 0 = control group, 1 = experimental group. *p < 0.05, **p < 0.01, ***p < 0.001.

**Table 2:** Regression analysis results on the impact of being the target of envy in Study 1.

Variable	Model 1	Model 2
Constant	2.61*** (0.06)	2.61*** (0.06)
Manipulation of perceived trust from leaders	0.30* (0.13)	0.30* (0.13)
Manipulation of perceived competitive climate	0.51*** (0.13)	0.51*** (0.13)
Perceived trust from leaders × perceived competitive climate manipulation (interaction)	–	0.56* (0.26)
*R*^2^	0.09	0.11
Δ*R*^2^	–	0.02*
*F*	10.57***	8.78***

*n* = 214; all coefficients are unstandardized, standard errors are reported in parentheses. *p < 0.05, **p < 0.01, ***p < 0.001.

**Figure 2:**
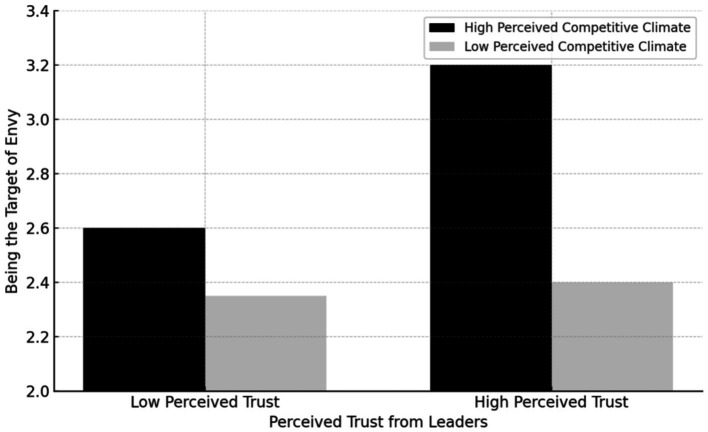
The moderating effect of perceived competitive climate on perceived trust and envy by leaders in Study 1.

As shown in Model 2 of Table 2, the interaction between Perceived trust from leaders and perceived competitive climate significantly influences envy (*b* = 0.56, SE = 0.26, *p* = 0.03). The moderating effect is shown in Figure 2. When the Perceived competitive climate was high, the envy scores of the high Perceived Trust from Leaders group (*M* = 3.18, SD = 0.89) were significantly higher than those of the low Perceived Trust from Leaders group (*M* = 2.59, SD = 0.98), *t* (103) = 3.20, *p* < 0.01, Cohen’s d = 0.63. When the Perceived competitive climate was low, the envy scores of the high Perceived Trust from Leaders group (*M* = 2.38, SD = 0.78) and the low Perceived Trust from Leaders group (*M* = 2.35, SD = 1.05) were not significantly different, *t* (107) = 0.15, *p* = 0.88, Cohen’s d = 0.03. Hypothesis 1 was confirmed.

In Study 1, our contextual experiment established a causal relationship between perceived trust from leaders and being the target of envy, with the effect amplified under a high Perceived competitive climate. The focal interaction remains significant (*p*s < 0.05), and effect sizes change minimally. These findings confirm the interactive influence of trust and perceived competitive climate on envy, supporting Hypothesis 1. However, the experimental setting limits the generalizability of these results to real-world workplaces. To address this, Study 2 employs a multi-source field survey to extend these findings to authentic organizational contexts, testing the full theoretical model and enhancing the external validity of the research.

## 4 Study 2

### 4.1 Research methods

#### 4.1.1 Research procedure and sample

Participants were recruited through alumni networks. This sampling method can recruit participants from different industries, thereby improving the external validity of the research conclusions (Qin et al., 2018). A total of 77 work teams were invited to participate in the survey. Each team consists of a single leader and multiple employees. Considering that in this survey, each leader must evaluate all employees under him/her, and each employee must evaluate other colleagues in the team, in order to reduce the burden on the respondents and ensure the quality of the answers, for teams with more than 10 people, 9 employees were randomly selected to participate in the survey, in addition to the leader. For the rest of the teams, all employees participated in the survey.

This study distributed electronic questionnaires using the Google Forms platform. Each work team established a Line group. To maintain ethical standards, participants were informed that all data would be anonymized, and their consent was obtained in advance. Before distributing the questionnaires, the researchers explained the purpose of the study in the group and emphasized that the results would be used solely for academic research and would not impact the participants. Furthermore, to ensure that team members’ perceived trust in their leaders was reflected in the same leader, the researchers confirmed the names of their team leaders and members in the Line group. After confirmation, the electronic questionnaire link was sent to the group. The electronic questionnaire consisted of two versions: a leadership version and an employee version. The leadership version was completed by team leaders, who were asked to rate the work performance of each participating employee. The employee version was completed by employees, who, in addition to providing demographic information, perceived trust from leaders, perceived competitive climate, and being the target of envy, were also asked to rate each colleague’s perceived workplace exclusion. For example, in a team of four participating employees, employee M1 was asked to rate M2, M3, and M4’s perceived workplace exclusion; employee M2 was asked to rate M1, M3, and M4’s perceived workplace exclusion, and so on. To motivate participants and ensure quality responses, each participant received a reward of 50 baht. Ultimately, 65 valid leadership data sets were collected (with an effective response rate of 84.4%) and 278 valid employee data sets (with an effective response rate of 89.1%). Of the 278 employees, 178 were male (64.0%) and 100 were female (36.0%). The average age was 32.99 years (SD = 7.88). In terms of educational background, 4.3% held a master’s degree or above, 39.2% held a bachelor’s degree, 30.2% held a college degree, and 26.3% held less than a college degree.

#### 4.1.2 Measurement tool

##### 4.1.2.1 Perceived trust from leaders

The Perceived Trust from Leaders Scale (PTS) by Baer et al. (2015) was used. It consists of 10 items, such as “My leader allows me to play a role in work that is important to him/her.” Employees were asked to answer based on their current feelings. The Cronbach’s α coefficient of this scale in this study was 0.85.

##### 4.1.2.2 Perceived competitive climate

The Perceived Competitive Climate Scale (PCO) by Brown et al. (1998) was used, consisting of four items (e.g., “My colleagues often compare their work performance with mine.”). Employees answered based on their current feelings. Reliability was acceptable in this study (α = 0.78). To establish validity, two subject-matter experts and five employees reviewed item clarity and relevance (face validity) prior to analysis. A preliminary EFA in Study 1 supported a single-factor solution. In Study 2, a four-factor CFA including perceived leader trust, perceived competitive climate, feeling envied, and job performance showed acceptable fit (χ^2^ = 452.35, df = 183; SRMR = 0.07; RMSEA = 0.07; CFI = 0.91; TLI = 0.90) and outperformed three-factor alternatives (e.g., merging feeling envied with perceived competitive climate: χ^2^ = 665.81, df = 186; Δχ^2^ = 213.46, *p* < 0.001), supporting construct validity. In Study 1, the manipulation check also showed high reliability (α = 0.95).

##### 4.1.2.3 Being the target of envy

The being the target of envy scale (PCO) by Lee et al. (2018) was used, consisting of three items, such as “Because of my success at work, I am sometimes envied by my colleagues.” Employees were asked to answer based on their current feelings. The Cronbach’s α coefficient for this scale in this study was 0.94.

##### 4.1.2.4 Workplace exclusion

Adapted from the workplace exclusion behavior scale developed by Yang and Treadway (2018), it consists of 1 item. A round-robin sampling method is used, with each employee evaluating the workplace exclusion behavior of other team members in turn. The question is: “In the past period of time, how often has this colleague excluded you? (For example, not responding to you when you greeted him, ignoring you, being indifferent to you, etc.).” Finally, each employee will receive the scores of other team members on their workplace exclusion behavior. The mean of these scores reflects the overall degree of workplace exclusion of the employee toward other team members.

##### 4.1.2.5 Control variables

Based on the relevant literature (Mao et al., 2021), we controlled for three employee demographics—gender, age, and educational background—to reduce potential alternative explanations. Because work performance is central to social comparison in organizations and a key antecedent of coworker envy (Kim and Glomb, 2014), we also controlled for employee performance using the four-item supervisor-rated scale developed by Morrison and Phelps (1999) (e.g., “The employee fully completes the assigned tasks.”), which showed high reliability in this study (Cronbach’s α = 0.92). To mitigate bias from stable trait-like predispositions (e.g., narcissism or neuroticism), the experiment relied on random assignment to balance such dispositions across conditions, and in all analyses we estimated extended-control specifications in which demographics and performance were entered on both the mediator and the outcome equations.

### 4.2 Research results

#### 4.2.1 Descriptive statistics and correlation analysis

The results of descriptive statistics and correlation analysis are shown in Table 3.

**Table 3:** Means, standard deviations, and correlation coefficients of variables in Study 2.

Variable	*M*	*SD*	1	2	3	4	5	6	7	8
1. Gender	0.36	0.48	–							
2. Age	1.76	0.79	−0.08	–						
3. Education level	2.22	0.89	0.06	−0.24***	–					
4. Job performance	4.34	0.59	0.05	−0.06	−0.02	–				
5. Perceived trust from leaders	3.89	0.65	−0.06	0.10	0.03	0.16**	–			
6. Perceived competitive climate	3.13	0.82	−0.09	0.18**	−0.13*	0.03	0.22***	–		
7. Being the target of envy	2.26	1.00	−0.13*	0.27***	−0.05	0.05	0.08	0.39***	–	
8. Workplace exclusion	1.15	0.37	−0.18**	−0.03	−0.04	0.03	−0.10	0.10	0.15*	–

*n* = 278; Gender: 0 = male, 1 = female; Age: 1 = 21–30 years, 2 = 31–40 years, 3 = 41–50 years, 4 = 51–60 years; Education level: 1 = below associate degree, 2 = associate degree, 3 = bachelor’s degree, 4 = master’s degree or above; *p < 0.05, **p < 0.01, ***p < 0.001.

#### 4.2.2 Confirmatory factor analysis

To examine the discriminant validity of the four variables of Perceived trust from leaders, perceived competitive climate, being the target of envy, and work performance, this study used Mplus 8.0 to conduct a confirmatory factor analysis. The results are shown in Table 4. As shown in Table 4, the fit index of the hypothesized four-factor model is significantly better than that of the other three three-factor models, indicating that the variables have good structural discriminability.

**Table 4:** Results of confirmatory factor analysis for Study 2.

Model description	χ^2^	df	Δχ^2^ (Δdf)	SRMR	RMSEA	CFI	TLI
Hypothesized model	452.35	183	–	0.07	0.07	0.91	0.90
Three-factor model (with being the target of envy and perceived competitive climate combined)	665.81	186	213.46 (3)	0.10	0.10	0.85	0.83
Three-factor model (with perceived trust from leaders and job performance combined)	1246.26	186	793.91 (3)	0.13	0.14	0.66	0.62
Three-factor model (with being the target of envy and job performance combined)	1283.21	186	830.86 (3)	0.14	0.15	0.65	0.60

*n* = 278. All Δχ^2^ were significant at the *p* < 0.001 level.

#### 4.2.3 Hypothesis testing

Considering that this study is a team sample, each team member evaluated the workplace exclusion behavior of multiple team members, and the team leader evaluated the work performance of multiple team members, so the data structure of this study is nested. To eliminate the problem of data non-independence, the statement “TYPE = COMPLEX; ESTIMATOR = MLR” was used in Mplus8.0 (Wu and Kwok, 2012). This method is suitable for exploring non-independent data of the relationship between variables at a single level (Wu and Kwok, 2012). However, the issues of this study are concentrated at the employee level, so this analysis method is suitable for this study.

**Figure 3:**
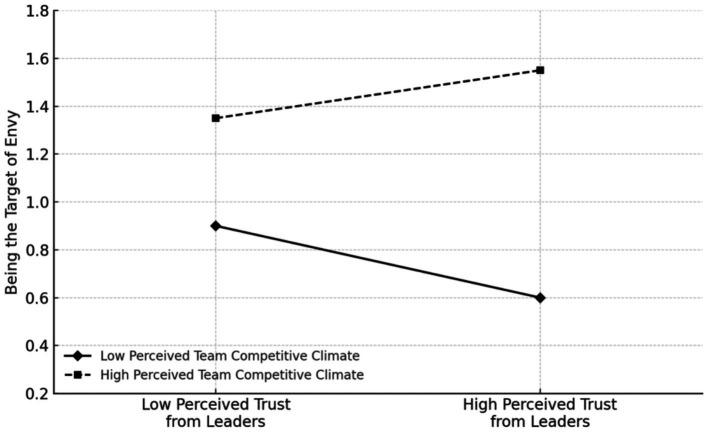
Study 2: The moderating effect of perceived competitive climate on the relationship between perceived trust and envy from leaders.

Hypothesis 1 posits that Perceived trust from leaders and perceived competitive climate interact to influence Being the target of envy. As shown in Model 4 of Table 5, the interaction term between Perceived trust from leaders and perceived competitive climate significantly impacts Being the target of envy (*b* = 0.21, SE = 0.08, *p* < 0.01). The simple effects plot is shown in Figure 3. These results indicate that the stronger the Perceived competitive climate, the stronger the positive impact of Perceived trust from leaders on Being the target of envy, confirming Hypothesis 1.

**Table 5:** Regression analysis results on the impact of being the target of envy in Study 2.

Variable	Model 1	Model 2	Model 3	Model 4
Constant	1.42** (0.43)	1.48** (0.44)	1.45** (0.42)	1.42** (0.42)
Gender	−0.24 (0.14)	−0.24 (0.14)	−0.19 (0.13)	−0.15 (0.13)
Age	0.34*** (0.07)	0.33*** (0.07)	0.27*** (0.07)	0.26*** (0.07)
Education level	0.03 (0.07)	0.03 (0.07)	0.07 (0.07)	0.06 (0.07)
Job performance	0.12 (0.09)	0.10 (0.09)	0.10 (0.09)	0.10 (0.08)
Perceived trust from leaders	–	0.06 (0.11)	−0.05 (0.10)	−0.05 (0.10)
Perceived competitive climate	–	–	0.43*** (0.08)	0.41*** (0.06)
Perceived trust from leaders × perceived competitive climate	–	–	–	0.21** (0.08)
*R*^2^	0.09	0.09	0.21	0.23

*n* = 278. All coefficients are unstandardized; standard errors are reported in parentheses. *p < 0.05, **p < 0.01, ***p < 0.001.

Hypothesis 2 states that being the target of envy is positively correlated with employees’ workplace exclusionary behavior toward colleagues. Table 6, Model 3, shows that after controlling for relevant variables, being the target of envy significantly and positively impacts employees’ workplace exclusionary behavior toward colleagues (*b* = 0.06, SE = 0.03, *p* = 0.02), confirming Hypothesis 2.

**Table 6:** Regression analysis results on the impact of workplace exclusion in Study 2.

Variable	Model 1	Model 2	Model 3
Constant	1.34*** (0.17)	1.28*** (0.15)	1.20*** (0.14)
Gender	−0.14* (0.06)	−0.15* (0.06)	−0.14* (0.06)
Age	−0.03 (0.03)	−0.02 (0.03)	−0.04 (0.03)
Education level	−0.02 (0.02)	−0.01 (0.02)	−0.02 (0.02)
Job performance	0.02 (0.03)	0.03 (0.03)	0.03 (0.03)
Perceived trust from leaders	–	−0.06 (0.05)	−0.07 (0.05)
Being the target of envy	–	–	0.06* (0.03)
*R*^2^	0.04	0.05	0.07

*n* = 278. All coefficients are unstandardized; standard errors are shown in parentheses. *p < 0.05, **p < 0.01, ***p < 0.001.

To test the moderated mediation hypothesis proposed in Hypothesis 3, according to the suggestions of Edwards and Lambert (2007), the indirect effects of the moderator variable under the conditions of one standard deviation higher and one standard deviation lower were calculated, and the difference in the indirect effects between the two conditions was tested to see whether it was significant.

Hypothesis 3 assumes that the Perceived competitive climate moderates the indirect effect of Perceived trust from leaders on workplace exclusion through envy. The results of Monte Carlo simulation (Selig and Preacher, 2008) show that when employees perceive a strong Perceived competitive climate, the indirect effect of Perceived trust from leaders on workplace exclusion through envy is 0.01, 95% CI = [−0.01, 0.03]; when employees perceive a weak Perceived competitive climate, the indirect effect of Perceived trust from leaders on workplace exclusion through envy is −0.01, 95% CI = [−0.04, 0.002]. The difference in the indirect effect between the two cases is 0.02, and 95% CI = [0.001, 0.05], excluding 0. Hypothesis 3 is confirmed. All hypothesized paths remained robust under extended controls.

## 5 Discussion

This study advances understanding of how leader trust can spill over to peer dynamics by acting as a visible status cue. We show that feeling envied increases for leader-trusted employees and that the envied target is more likely to engage in exclusionary behavior toward coworkers as a defensive coping response. These relationships remain robust under extended-control specifications.

We explicitly distinguish workplace ostracism from the outcome studied here. Workplace ostracism is other-directed mistreatment in which the focal employee perceives being ignored or excluded by coworkers (Mayer et al., 1995; Sun et al., 2020; Koopman et al., 2020). Our outcome is the envied target’s exclusionary behavior, namely target-initiated withdrawal or avoidance in response to feeling envied. Keeping these constructs separate prevents contamination between being excluded by others and choosing to distance oneself.

### 5.1 Theoretical contributions

This study has important theoretical implications for the study of trust, envy, and workplace ostracism.

First, this study refines the research perspective on the outcome variable of Perceived trust from leaders, helping researchers gain a more comprehensive and balanced understanding of the impact of perceived trust from leaders within organizations. Existing research has demonstrated the impact of Perceived Trust from Leaders on employees and their relationships with leaders from the perspective of both employees themselves and the relationship between employees and leaders (Wang and Zhang, 2016; Baer et al., 2015; Chen et al., 2020; Deng and Wang, 2009). Although there is evidence that the trust relationship between employees and leaders is also closely related to the interests of other colleagues in the organization (Graen and Uhl-Bien, 1995; Sun et al., 2020), existing research has not explored the impact of perceived trust from leaders from this perspective. This study, from the perspective of employee-colleague relationships, found that perceived trust from leaders may lead employees to feel jealous, which in turn leads them to engage in workplace exclusionary behaviors toward their colleagues. At the same time, the competitive climate perceived by employees within the team will enhance the above effects. From the perspective of social comparison theory, this result provides an explanation for why, how, and when Perceived Trust from Leaders affects the relationship between employees and colleagues. It not only provides a new perspective for the study of the outcome variables of perceived trust from leaders, but also responds to Baer et al.’s (2015) call for strengthening the exploration of the important mechanism of perceived trust from leaders.

Second, this study enriches the research on the “dark side” of Perceived Trust from Leaders and provides a more dialectical perspective for understanding the role of Perceived Trust from Leaders. The vast majority of existing studies show that perceived trust from leaders will have a positive impact (Lau et al., 2014). Only a few studies have explored the possible negative impact of perceived trust from leaders from the perspectives of stress and self-concept (Wang and Zhang, 2016; Baer et al., 2015; Chen et al., 2020). This study, from the perspective of social comparison, found the possible negative impact of perceived trust from leaders on colleagues. This result not only further challenges the mainstream view that “trust is always beneficial,” allowing researchers to understand the role of perceived trust from leaders more comprehensively and dialectically, but also responds to the call of Chen et al. (2020) to further explore the potential negative consequences of perceived trust from leaders. Third, this study expands the research on the causes and consequences of being the target of envy. Existing studies have mainly focused on issues related to envy, with less attention paid to being the target of envy. According to the definitions of researchers, the definitions of envy and being the target of envy are different (Liu et al., 2018). Although some researchers have pointed out that Being the target of envy may be affected by leadership and organizational factors (Liu et al., 2018), except for Ng (2017), there are currently few studies that directly analyze and verify which factors in the organization affect individuals’ feelings of Being the target of envy. Similarly, because being the target of envy has only recently begun to attract researchers’ attention, researchers currently know very little about the impact of being the target of envy in organizations. This study uses envy as a mediating variable to explore the relationship between factors such as trust in leadership and workplace exclusion and envy. The results will further enrich the antecedents and consequences of being the target of envy and promote the development of envy research.

Fourth, this study enriches the research on the antecedents of workplace exclusion from the perspective of the excluder. Currently, most research on the antecedents of workplace exclusion in the field of organizational management explores “what kind of employees are more likely to be excluded in the workplace” from the perspective of the excluded (Wang et al., 2021), while research exploring “why individuals exclude others” from the perspective of the excluder is relatively lacking (Chen et al., 2017). This study found that in situations where employees perceive a strong Perceived competitive climate, Perceived trust from leaders may also indirectly trigger employees’ workplace exclusion behavior toward colleagues through envy. This finding further enriches the research on the antecedents of workplace exclusion from the perspective of the excluder and explains why employees exclude others from the workplace from a social comparison perspective.

### 5.2 Practical implications

This study provides the following three main implications for leaders:

First, in the process of trust management, leaders should fully understand the potential impact of trust on employees. Although much evidence shows that Perceived trust from leaders is a recognition of employees’ roles and status in the organization, which can motivate employees’ work enthusiasm and improve their work performance (Wei and Long, 2009), the results of this study indicate that the perception of leader trust may also increase employees’ being envied and workplace exclusion of colleagues. When leaders only trust a very small number of subordinates, the scarcity of Perceived trust from leaders will lead to increased social comparison intensity among colleagues (Festinger, 1954), thereby leading to unhealthy competition and exclusion among team members, and ultimately hindering the long-term healthy development of the organization. Therefore, leaders need to avoid trusting only a very small number of subordinates as much as possible. When managing team trust, they can choose different trust strategies to try to meet the trust needs of each subordinate.

Second, leaders need to pay attention to the Perceived competitive climate in the team and promote moderate and healthy competition. Although a Perceived competitive climate can bring about a “catfish effect,” making employees feel threatened and thus motivating them, this study found that a highly Perceived competitive climate may also intensify envy and exclusion among colleagues. Therefore, leaders need to pay attention to the overall Perceived competitive climate of the team and avoid the negative effects caused by an overly Perceived competitive climate.

Third, leaders should pay more attention to and guide employees’ mental health, guide employees to make moderate social comparisons, and rationally view the differences between themselves and others. Social comparison is a common social psychological phenomenon that helps individuals understand and evaluate themselves (Festinger, 1954). This study found that excessive social comparison among colleagues can lead to envy and workplace exclusion among colleagues, which is not conducive to team cooperation and development. Therefore, leaders can guide employees to make social comparisons with a positive attitude by offering relevant mental health training lectures, give full play to the positive social function of social comparison (Liu et al., 2017), and avoid employees from developing extreme emotions and behaviors.

This study was conducted in Thailand, where collectivist cultural norms emphasize group harmony and interdependence. In such a context, envy may be more pronounced due to group-oriented norms that heighten sensitivity to social comparisons and perceived inequities, potentially amplifying exclusionary behaviors among coworkers. Employees who are trusted by leaders may stand out as deviating from group norms, making them more likely to be envied and, consequently, to engage in exclusionary behaviors as a defensive response. This cultural dynamic suggests that the effects of perceived trust on envy and workplace exclusion may vary across cultural settings. Future research could explore these dynamics in individualistic cultures, where personal achievement is prioritized, to assess the generalizability of our findings and further elucidate the role of cultural context in shaping workplace interactions.

### 5.3 Research deficiencies and future research prospects

This study has the following deficiencies, which can be further explored and improved in future research.

First, although Study 2 of this paper avoided possible common method bias by adopting a multi-source sampling method, and the verification of the moderating effect of Perceived competitive climate also showed that there was no serious common method bias in Study 2 (Podsakoff et al., 2012), since the data of the relevant variables in Study 2 were collected at the same time point, may introduce potential inflation bias between the variables. Therefore, future research can consider using a multi-time point method to collect data. In addition, although Study 2 controlled employees’ demographic characteristics and work performance with reference to existing research, existing research shows that in addition to these factors, some individual factors (such as narcissism) may also affect envy (Koopman et al., 2020). Future research needs to control these variables to exclude possible alternative explanations and further enhance the causal relationship judgment of the research.

Second, this study explored how Perceived trust from leaders affects the relationship between employees and colleagues from the perspective of social comparison theory. Future research can explore this issue from more theoretical perspectives. For example, based on balance theory (Heider, 1958), when two employees in the same team perceive different levels of trust from their leaders, this may lead to an imbalance in their interactions, which in turn affects their relationships and behaviors. Based on extended self theory (Wang et al., 2019), employees who perceive trust from their leaders may want to claim the trust from their leaders as their own, developing a psychological ownership of the trust from their leaders, thereby engaging in territorial behavior toward their colleagues to prevent their colleagues from competing with them for the trust from their leaders.

Third, based on social comparison theory, this study selected employee-perceived team climate as the boundary condition for the effect of perceived trust from their leaders on the interactions between employees and their colleagues. Future research could further explore the moderating role of other factors (e.g., employee and leader factors). For example, employee narcissism may be a boundary condition for the effect of perceived trust from their leaders on the interactions between employees and their colleagues. Individuals with high narcissism are more focused and obsessed with their own image and are more likely to exaggerate their own importance and superiority (Yang and Zhang, 2021). Therefore, when high narcissistic employees perceive that they are trusted by their leaders, they are more likely to feel jealous and thus exhibit more workplace exclusion behaviors toward their colleagues. Leadership power may also moderate the impact of perceived trust from leaders on being the target of envy and workplace exclusion. When leadership power is strong, employees who gain the trust of their leaders may gain greater benefits in the organization (Wang et al., 2018) and are also more likely to be envied by their colleagues and exhibit more workplace exclusion behaviors. Conversely, when leadership power is weak, even if employees gain the trust of their leaders, it is difficult for them to gain actual benefits in the organization. Therefore, they do not need to worry about being the target of envy and will not exhibit workplace exclusion behaviors.

## Data Availability

The original contributions presented in the study are included in the article/supplementary material, further inquiries can be directed to the corresponding author.
